# Highlight: Genetic Signatures of Chickpea's Cultural Crossroads

**DOI:** 10.1093/molbev/msad135

**Published:** 2023-06-22

**Authors:** Casey McGrath

With its nutty flavor and dense nutrient profile, the humble chickpea has captivated palates and nourished civilizations for millennia. From its ancient origins to its widespread use in modern kitchens and restaurants around the world, this legume demonstrates both culinary versatility and cultural significance. Despite prominence in traditional cuisines across several continents, the origin, diversification, and spread of chickpeas throughout the Middle East, South Asia, Ethiopia, and the western Mediterranean have remained a mystery. A new study in *Molecular Biology and Evolution* titled “Historical routes for diversification of domesticated chickpea inferred from landrace genomics” (Igolkina et al. 2023) sheds light on the profound effects of human migration and trade on chickpea's genetic heritage.

**Fig. 1.**
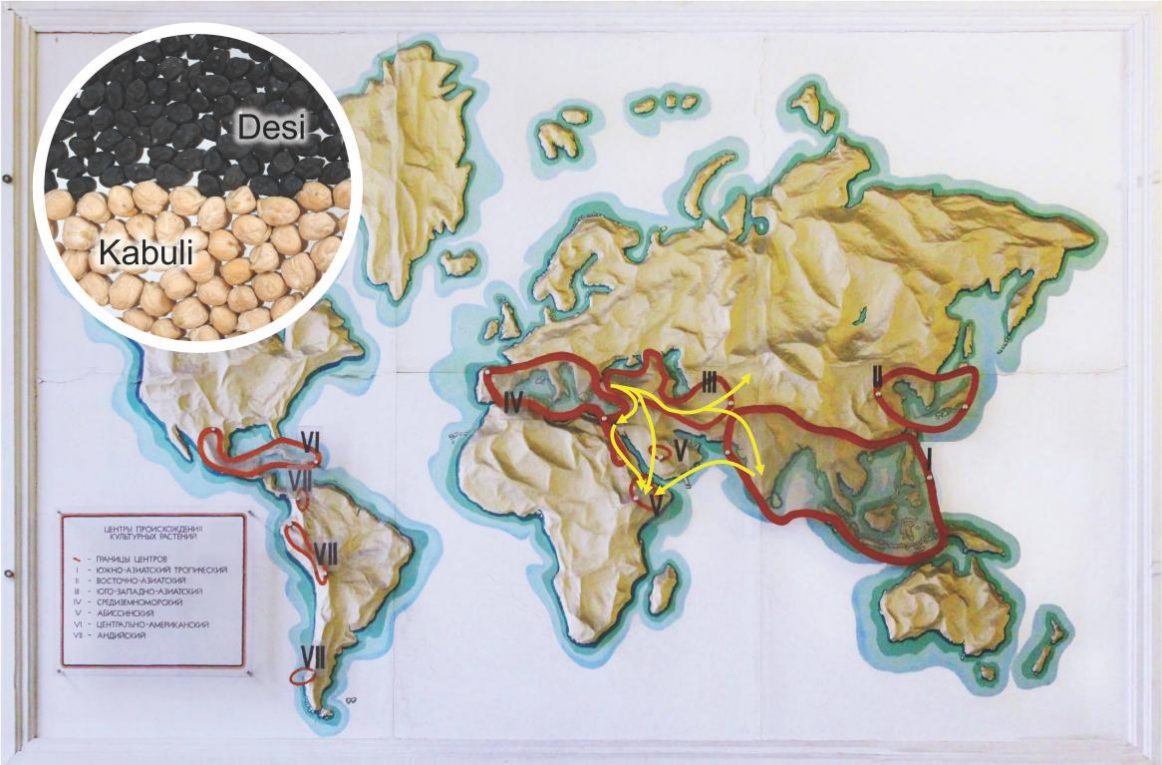
A map derived from Nikolai Vavilov's original depiction of chickpea domestication centers (Roman numerals) showing chickpea dispersal routes (arrows) from its center of domestication in Southwest Asia to South and Central Asia and East Africa. Inset shows the desi and kabuli market classes of chickpea.

The study—led by Anna Igolkina from Peter the Great St. Petersburg Polytechnic University, Eric von Wettberg from the University of Vermont, and Sergey Nuzhdin from the University of Southern California—used genetic data from over 400 chickpea specimens collected in the 1920s and 1930s. The collection included both the desi and kabuli subtypes, which differ in color and size, despite the lack of a clear geographic or genetic boundary between the two (fig. 1). Chickpea samples came from nine different geographic regions: northern Mediterranean, southern Mediterranean, Turkey, Lebanon, Ethiopia, the Black Sea, western Uzbekistan, eastern Uzbekistan, and India. To analyze the data, the researchers developed two new models, which they named popdisp (population dispersals) and migadmi (migrations and admixtures).

The authors used the popdisp model to understand how chickpeas dispersed within each of the geographic regions. They compared two scenarios, one in which chickpeas spread along routes that were easier for humans to traverse (i.e., possible historic trade routes) and one in which chickpeas dispersed over distances in a simple linear fashion, regardless of intervening geographic barriers. According to Igolkina, “Our study reveals an intriguing finding regarding the genetic relatedness among chickpea landraces in different geographic regions. Contrary to the assumption that genetic similarity would be determined by linear distance, our results suggest that it is more influenced by human movement costs. This implies that the spread of chickpea within each region occurred predominantly along trade routes, rather than through simple diffusion.”

Using the migadmi model, the scientists sought to uncover the origin of the Ethiopian desi population. “Ethiopian chickpeas have a unique flavor,” says von Wettberg, “with the tartness of the black desi chickpeas that can be found in Indian varieties, but also a hint of sweetness.” Previous studies have suggested two possible origins for Ethiopian chickpeas—either an Indian origin supported by morphological similarities or a Middle Eastern origin given the evidence of human migration from western Eurasia into East Africa around 4,500 years ago. Interestingly, the results revealed that both scenarios may be true, finding that Ethiopian chickpeas share ancestry from Indian, Lebanese, and Black Sea source populations. von Wettberg notes “For me, the most exciting finding is that Ethiopian chickpeas are a mixture of Middle Eastern and South Asian ancestry. The cultural connection of Ethiopians to the Middle East is widely known, exemplified by their Semitic heritage. Less well known is the extent and importance of Indian Ocean trade routes, which were both an important maritime route of the Silk Road and a way by which agricultural and cultural exchange happened between South Asia and East Africa.”

The migadmi model also revealed the possible origin of the kabuli type from a local desi chickpea population in Turkey. This disputes the linguistic suggestion that the kabuli type arose in Central Asia and is named after Kabul city (in modern Afghanistan).

While these results provide a fascinating look into the chickpea's natural history and its interconnectedness with human trade routes and migrations, the implications of this study extend far beyond chickpeas alone. “The importance of this work lies not only in extending our knowledge of chickpea history but also in the development of the two new models, popdisp and migadmi,” notes Igolkina. “These models can be applied together or separately to analyze migrations and admixtures in other species. The core modeling technique used in these models, compositional data analysis, allows for their extension to model multiallelic genetic markers. This is of particular interest when analyzing structural variants, analyses of which are becoming increasingly common.” von Wettberg concurs “A central part of our work is developing new tools for examining complex migration patterns. We hope others will use these tools to investigate similar migration patterns, either in human-associated species like crops, pests, and mutualists, or in natural species.”
